# Effect of Telmisartan or Losartan for Treatment of Nonalcoholic Fatty Liver Disease: Fatty Liver Protection Trial by Telmisartan or Losartan Study (FANTASY)

**DOI:** 10.1155/2013/587140

**Published:** 2013-08-13

**Authors:** Takumi Hirata, Kengo Tomita, Toshihide Kawai, Hirokazu Yokoyama, Akira Shimada, Masahiro Kikuchi, Hiroshi Hirose, Hirotoshi Ebinuma, Junichiro Irie, Keisuke Ojiro, Yoichi Oikawa, Hidetsugu Saito, Hiroshi Itoh, Toshifumi Hibi

**Affiliations:** ^1^Department of Internal Medicine, School of Medicine, Keio University, 35 Shinanomachi, Shinjuku-ku, Tokyo 160-8582, Japan; ^2^Department of Internal Medicine, National Defense Medical College, 3-2 Namiki, Tokorozawa-Shi, Saitama 359-8513, Japan

## Abstract

*Aim.* This study compared the effects of telmisartan and losartan on nonalcoholic fatty liver disease (NAFLD) and biochemical markers of insulin resistance in hypertensive NAFLD patients with type 2 diabetes mellitus. *Methods.* This was a randomized, open-label, parallel-group comparison of therapy with telmisartan or losartan. Nineteen hypertensive NAFLD patients with type 2 diabetes were randomly assigned to receive telmisartan at a dose of 20 mg once a day (*n* = 12) or losartan at a dose of 50 mg once a day (*n* = 7) for 12 months. Body fat area as determined by CT scanning and hepatic fat content based on the liver-to-spleen (L/S) ratio, as well as several parameters of glycemic and lipid metabolism, were compared before and after 12 months. *Results.* The telmisartan group showed a significant decline in serum free fatty acid (FFA) level (from 0.87 ± 0.26 to 0.59 ± 0.22 mEq/L (mean ± SD), *P* = 0.005) and a significant increase in L/S ratio (*P* = 0.049) evaluated by CT scan, while these parameters were not changed in the losartan group. *Conclusion.* Although there was no significant difference in improvement in liver enzymes with telmisartan and losartan treatment in hypertensive NAFLD patients with type 2 diabetes after 12 months, it is suggested that telmisartan may exert beneficial effects by improving fatty liver.

## 1. Introduction

Nonalcoholic fatty liver disease (NAFLD) is one of the most common forms of chronic liver disease throughout the world [1]. NAFLD is characterized by hepatic steatosis in the absence of significant alcohol use, hepatotoxic medication, or other known liver diseases [2]. NAFLD represents a spectrum ranging from simple fatty liver to nonalcoholic steatohepatitis (NASH), which is an aggressive form of NAFLD leading to cirrhosis and hepatocellular carcinoma [3–6]. Recently, it has been established that NAFLD is commonly associated with metabolic syndrome, including type 2 diabetes, obesity, dyslipidemia, and hypertension and consequently is associated with cardiovascular mortality [6–10]. The potential need for treatment of NAFLD is recognized, in order to improve cardiovascular and liver-related outcomes, and several therapeutic interventions to treat various components of metabolic syndrome have been evaluated [10–12].

Angiotensin II receptor blockers (ARBs), which are highly selective for the angiotensin II type 1 (AT1) receptor and block diverse effects of angiotensin II, are commonly used to treat hypertension [13]. Recently, ARBs have been expected to be effective for treatment of NAFLD, due to targeting of the mechanisms of insulin resistance and hepatic injury via suppression of the renin-angiotensin system (RAS), which has been suggested to be involved in the pathways of liver damage. It has been reported that an ARB, losartan, showed significant improvement in aminotransferase levels and serum markers of fibrosis in hypertensive patients with NASH [14]. Moreover, losartan has been reported to decrease the number of activated hepatic stellate cells, which play a pivotal role in the progression of hepatic fibrosis [15]. These results suggest that losartan might be therapeutically efficacious for NASH.

Telmisartan, another ARB, has been reported to have a partial agonistic effect on peroxisome proliferator-activated receptor (PPAR)-*γ* in addition to the effect of angiotensin II blockade [16, 17]. So, telmisartan is expected to have more potent effects in NAFLD than those of losartan, via PPAR*γ* activation, which promotes hepatic fatty acid oxidation, decreases hepatic lipogenesis, and increases peripheral and hepatic insulin sensitivity [18, 19]. In fact, it is reported that telmisartan attenuated steatohepatitis progression in an animal model [20]. In addition, telmisartan has been reported to improve insulin resistance and liver injury, based on measurement of homeostasis model assessment-insulin resistance (HOMA-IR) and serum aminotransferase (ALT) levels in humans [21].

In the present study, we tested the hypothesis that telmisartan might have a more potent effect on NAFLD and biochemical markers of insulin resistance than does losartan.

## 2. Materials and Methods

This study was conducted in accordance with the Declaration of Helsinki and was approved by the Ethics Review Board of Keio University. Written informed consent was obtained from each subject before participation in the study. This study was assigned the UMIN-ID, UMIN000000540.

### 2.1. Subjects

We screened patients with type 2 diabetes between 20 to 80 years of age with both NAFLD and hypertension. NAFLD was defined as fatty liver on ultrasonography, and aspartate aminotransferase (AST) level over 30 IU/L, and/or alanine aminotransferase (ALT) level over 40 IU/L. A detailed history of alcohol consumption was taken by physicians. All patients consumed less than 20 g of pure alcohol per day, and were negative for hepatitis B serological tests, antibody to hepatitis C virus, and autoantibodies, including anti-mitochondrial antibody and anti-nuclear antibody. Hypertension was defined as systolic blood pressure (SBP) over 140 mmHg and/or diastolic blood pressure (DBP) over 90 mmHg. Patients using antihypertensive agents were also included. Exclusion criteria included the presence of AST > 100 IU/L and/or ALT > 100 IU/L, severe hypertension (i.e., SBP > 200 mmHg, DBP > 120 mmHg), malignancy and recent major macrovascular disease (i.e., cardiovascular disease or stroke within past 3 months), insulin, biguanide or thiazolidinedione treatment for diabetes mellitus, and drug allergy to ARBs.

### 2.2. Study Design

This was a randomized, open-label, parallel-group comparison of therapy with telmisartan or losartan. Nineteen hypertensive NAFLD patients with type 2 diabetes were randomly assigned to the telmisartan (T) group (receiving a standard dose of 20 mg once daily, *n* = 12) or losartan (L) group (receiving a standard dose of 50 mg once daily, *n* = 7). Patients using other antihypertensive agents were randomly switched to telmisartan or losartan. Medication was not masked, and treatment had to be taken daily at the same hour in the morning, with no concomitant medication or alcohol consumption allowed. Either the patient or the medical staff was aware of the treatment group allocation. All 19 subjects received dietary instructions using a meal-exchange plan from nutritionists. The ideal dietary caloric intake for each patient was calculated as the ideal body weight (kg) × 25 kcal/kg. It was confirmed by questionnaire that the physical activity level was almost constant in each subject throughout the study period.

The included patients were followed for 12 months, with two-monthly visits. 

Anthropometric measurements, blood pressure (BP), heart rate (HR), and several clinical and biochemical parameters of glycemic control, lipid metabolism, and liver function were checked at every visit. Body fat area as determined by computed tomographic (CT) scanning at the umbilical level, hepatic fat content based on the liver-to-spleen (L/S) ratio according to CT attenuation values, inflammatory markers, and serum bile acid level were determined before and after 12 months.

### 2.3. Measurements

Blood pressure was determined in the sitting position after a 10-minute rest. Body weight was measured at the clinic under the same conditions for each patient. Blood samples were taken from each subject before breakfast in the early morning, after overnight bed rest.

Fasting plasma glucose (FPG) was determined by the glucose oxidase method. Hemoglobin A1c (HbA1c) was determined by high-performance liquid chromatography (Toso, Tokyo, Japan) and presented as the equivalent value for the National Glycohemoglobin Standardization Program (NGSP). Serum immunoreactive insulin (IRI) was measured by an enzyme immunoassay using a commercially available kit. Homeostasis model assessment-insulin resistance (HOMA-IR) was calculated by the formula: fasting plasma insulin (*μ*U/mL) × fasting plasma glucose (mg/dL)/405. HOMA-*β* was calculated by the formula: fasting plasma insulin (*μ*U/mL) × 360/(fasting plasma glucose (mg/dL) − 63) [22]. Total cholesterol (TC), high-density lipoprotein cholesterol (HDL-C), triglyceride (TG), and free fatty acids (FFAs) were measured enzymatically by an autoanalyzer (Hitachi, Tokyo, Japan). As biochemical parameters, AST, ALT, gamma glutamyl transpeptidase (*γ*GT), alkaline phosphatase (ALP), lactate dehydrogenase (LDH), blood urea nitrogen (BUN), creatinine (CR), uric acid (UA), sodium (Na), potassium (K), ferritin, and creatine phosphokinase (CPK) were measured. Inflammatory markers such as hyaluronic acid (Hyal), 7S domain of type IV collagen (4Col7S), high-sensitivity C-reactive protein (hs-CRP), procollagen III peptide (P-3-P), zinc (Zn), total adiponectin, and interleukin (IL)-6 were analyzed at the Special Reference Laboratory (SRL, Tokyo, Japan). We also measured bile acid (BA) components by high-performance liquid chromatography, because BAs might be related to lipid absorption and cholesterol catabolism [23].

Subcutaneous and visceral fat distribution was determined by measuring a −150 Hounsfield unit (HU) to −50 HU area using the method of CT scanning at the umbilical level as described previously [24]. V/S ratio was calculated as visceral fat area (VFA)/subcutaneous fat area (SFA). An index of fat deposition in the liver based on the liver-to-spleen (L/S) ratio according to CT attenuation values was also determined. The mean HU values of the liver and spleen were determined in the parenchyma of the right (CT-L1) and left lobe (CT-L2) of the liver and approximately the same size area of the spleen (CT-Spleen), avoiding blood vessels, artifacts, and heterogeneous areas. L/S ratio was calculated as [((CT-L1) + (CT-L2))/2]/(CT-Spleen).

### 2.4. Statistical Analyses

Continuous variables are presented as mean ± standard deviation. Continuous variables were compared between the telmisartan group and losartan group using the Mann-Whitney *U* test for independent samples. Differences in each baseline treatment between groups were analyzed by chi-squared test. Differences in each parameter between the start and after 12 months in each group were analyzed using the Wilcoxon's matched-pair signed-rank test. A *P* value less than 0.05 was considered to be statistically significant. Statistical analyzes were carried out using StatView 5.0 software (SAS Institute, Cary, NC, USA).

## 3. Results

### 3.1. Baseline Characteristics

Baseline characteristics of the subjects in both groups are shown in Table 1. There were no significant differences in most parameters including duration of diabetes, anthropometric measurements, BP, biochemical measurements, and inflammatory markers between the T group and L group. In spite of randomization, there were significant differences in two parameters between the two groups at baseline; serum FFA (0.87 ± 0.26 mEq/L in T group versus 0.50 ± 0.26 mEq/L in L group (*P* = 0.001)) and L/S ratio (0.82 ± 0.25 in T group versus 1.01 ± 0.23 in L group (*P* = 0.035)).

### 3.2. Changes in Anthropometric Measurements and BP

No subject terminated the trial because of adverse events. 

Body weight and waist and hip measurements did not change in both groups. Both groups showed a significant decrease in SBP (139.4 ± 11.1 versus 130.8 ± 15.0 mmHg in T group (*P* = 0.045), 136.4 ± 13.9 versus 127.4 ± 10.6 mmHg in L group (*P* = 0.046)) after 12 months. Concerning DBP, a statistically significant decrease was found in the T group (86.0 ± 8.5 versus 75.4 ± 12.7 mmHg (*P* = 0.032)), whereas the decrease in the L group did not reach statistical significance (81.6 ± 13.0 versus 75.3 ± 7.9 mmHg (*P* = 0.116)) (Table 2).

### 3.3. Changes in Biochemical Measurements

Liver enzyme levels such as AST, ALT, and *γ*GT did not show significant change in both groups after 12 months. While TC, HDL-C, and TG levels did not show significant change in both groups after 12 months, FFA level showed a significant decrease in the T group (0.87 ± 0.26 versus 0.59 ± 0.22 mEq/L (*P* = 0.005)) whereas the change in the L group was not significant (0.50 ± 0.26 versus 0.66 ± 0.22 mEq/L (*P* = 0.237)).

FPG level did not change in both groups after 12 months. Regarding HbA1c level, the L group showed a significant increase (6.7 ± 1.0 versus 7.2 ± 1.2% (*P* = 0.017)), while the change in the T group was not significant (6.4 ± 0.6 versus 6.4 ± 0.4% (*P* = 0.552)).

UA level showed a significant decrease in the L group (5.7 ± 1.5 versus 5.2 ± 1.3 mg/dL (*P* = 0.046)), while it showed a significant increase in the T group (5.8 ± 1.4 versus 6.3 ± 1.2 mg/dL (*P* = 0.016)). Consequently, the difference in changes was also statistically significant.

Levels of other inflammatory markers and bile acids did not show significant change in both groups after 12 months (Tables 3 and 4).

### 3.4. Changes in Fat Distribution and Fat Deposition in Liver

Visceral and subcutaneous fat area did not change in both groups after 12 months. Consequently, V/S ratio did not change in both groups. Regarding L/S ratio, a significant increase was found in the T group (0.82 ± 0.25 versus 0.97 ± 0.22 (*P* = 0.049)), while it did not change in the L group (1.01 ± 0.23 versus 1.01 ± 0.21 (*P* > 0.999)) (Table 5).

## 4. Discussion

In the present study, we evaluated the effects of ARBs (telmisartan and losartan) on NAFLD in hypertensive patients with type 2 diabetes and compared their effect to improve liver function after 12 months of treatment.

There was no significant improvement in liver function in either group. However, serum FFA level was significantly decreased in the telmisartan group, leading to a significant improvement in L/S ratio, which reflects the severity of fatty change in the liver, compared to that in the losartan group. This finding suggests that telmisartan might improve fat deposition in the liver.

Unlike other ARBs, telmisartan is known to activate PPAR*γ* [16, 17, 25]. Its activation induces insulin sensitization through an increase in adiponectin in adipose tissue. In fact, several reports have been published concerning the efficacy of the PPAR*γ* agonist, pioglitazone, in the treatment of NASH. It is known that pioglitazone improves liver histological features, including steatosis, hepatocellular ballooning degeneration, lobular inflammation, and fibrosis [26–28]. In studies using several strains of animal models, telmisartan inhibited fat deposition, inflammation, and fibrosis in the liver [20, 29–31]. Also, these effects of telmisartan were greater than those of another ARB, valsartan [32], with the expectation of its efficacy in the liver also in humans.

In the present study, liver enzyme levels were not significantly improved in either the telmisartan or losartan group over 12 months. In a previous study, 48-week treatment with losartan significantly improved liver enzyme levels [14]. However, liver enzyme levels before ARB administration in the study were higher compared with those in our study, and after one year of administration of ARB they were only reduced to around the same levels as found in our study. Because the subjects had mild high levels of liver enzymes in the present study, it might have been difficult to observe marked improvement of liver enzyme levels.

It is notable that there was a significant decrease in serum FFA level in the telmisartan group compared to that in the losartan group in the present study. Reduction in serum FFA can improve insulin resistance and reduce fat deposition in the liver as ectopic fat [33, 34]. Here, the L/S ratio, which indicates fat deposition in the liver [35], was significantly increased in the telmisartan group but not in the losartan group, suggesting that this might be associated with the ability of telmisartan to activate PPAR*γ* [16, 17]. However, in the losartan group, a low serum FFA level and a L/S ratio were found at baseline compared to those in the telmisartan group, suggesting low insulin resistance and less fat deposition in the liver. Thus, this suggests that it would not be possible to observe improvement of FFA and L/S ratio in the losartan group. In addition, glycemic control deteriorated over 12 months in the losartan group.

It is reported that telmisartan, but not losartan, displayed insulin-sensitizing activity in a clinical study, which may be explained by its partial PPAR*γ* activity [36]. Telmisartan might have a preventive effect against progressive deterioration of beta-cell function.

Although a few clinical trials have examined the effects of ARBs on NAFLD, they were mostly conducted in patients with NAFLD with markedly elevated liver enzyme levels. However, epidemiologic studies conducted in Japan showed that liver enzyme levels remained only slightly elevated in many patients [37]. The present study included patients with NAFLD, which is often seen in daily clinical practice, and thus was meaningful in regard to examining the effect of ARBs in a more realistic setting. Furthermore, the present study is thought to be meaningful since the effects of telmisartan and losartan in treating NAFLD have not been examined in a randomized controlled study.

Our study has several limitations. First, the small number of patients and the deviation between groups in spite of randomization made it difficult to detect differences in outcomes between groups. Especially, differences in BMI and duration of diabetes between groups might affect the results. Therefore, randomized controlled studies with larger numbers of patients might be needed in the future. Secondly, in this study, dietary instruction and exercise therapy were left entirely to the discretion of the outpatient attending physicians rather than implementing specific patient education programs. For this reason, although it was uncertain whether dietary and exercise therapy were sufficient or not in either group, an increase in BMI was not observed at least in the telmisartan group, suggesting that dietary and exercise therapy were probably sufficient. Lastly, we did not perform histological examination of fat deposition, inflammation, or fibrosis in the liver. It is thus unclear whether histological changes occurred in the liver tissue due to treatment with either drug.

## 5. Conclusion

In this randomized controlled study that examined the effect of telmisartan and losartan in improving steatosis in hypertensive NAFLD patients with type 2 diabetes, significant improvement in liver function was not observed in either group. However, serum FFA level was significantly reduced in the telmisartan group compared to the losartan group. In addition, unlike losartan, telmisartan improved the L/S ratio. Due to its potential to improve fat deposition in the liver, telmisartan could be a therapeutic option in the treatment of NAFLD. In the future, a large-scale clinical study is needed to determine the utility of telmisartan in the treatment of NAFLD.

## Figures and Tables

**Table 1:** Baseline characteristics in each group.

Parameters	Telmisartan	Losartan	*P*-value
*N* (male/female)	**12 (6/6)**	**7 (3/4)**	
Age (years)	57.7 ± 12.8	60.3 ± 14.3	0.612
Duration of diabetes (years)	5.4 ± 6.0	6.1 ± 6.9	0.523
Height (cm)	161.8 ± 11.0	158.9 ± 13.5	0.673
Body mass index (kg/m^2^)	29.2 ± 5.8	27.8 ± 3.8	0.735
Waist circumference (cm)	97.0 ± 14.9	94.4 ± 8.1	0.899
Hip circumference (cm)	111.9 ± 20.9	99.6 ± 8.7	0.257
Systolic blood pressure (mmHg)	139.4 ± 11.1	136.4 ± 13.9	0.526
Diastolic blood pressure (mmHg)	86.0 ± 8.5	81.6 ± 13.0	0.611
Pulse (beats/min)	74.5 ± 11.8	85.0 ± 9.5	0.205
Biochemical markers			
Fasting plasma glucose (mg/dL)	116.5 ± 20.3	122.8 ± 20.2	0.571
Hemoglobin A1c (%)	6.4 ± 0.6	6.7 ± 1.0	0.444
Glycoalbumin (%)	15.9 ± 2.6	17.0 ± 3.0	0.447
Immunoreactive insulin (*μ*U/mL)	12.5 ± 6.1	12.6 ± 6.4	0.955
Total cholesterol (mg/dL)	217.8 ± 42.3	201.0 ± 38.9	0.447
High-density lipoprotein cholesterol (mg/dL)	52.8 ± 13.1	46.7 ± 9.2	0.290
Triglyceride (mg/dL)	122.3 ± 54.3	120.9 ± 45.7	0.866
Free fatty acids (mEq/L)	0.87 ± 0.26	0.50 ± 0.26	0.001
Aspartate aminotransferase (IU/L)	30.6 ± 13.9	32.0 ± 10.3	0.372
Alanine aminotransferase (IU/L)	39.8 ± 26.6	43.7 ± 26.2	0.583
*γ* Glutamyl transpeptidase (IU/L)	58.9 ± 43.0	60.9 ± 63.8	0.612
Alkaline phosphatase (IU/L)	249.2 ± 56.9	256.9 ± 97.0	0.800
Lactate dehydrogenase (IU/L)	193.4 ± 23.7	207.6 ± 40.3	0.353
Blood urea nitrogen (mg/dL)	13.5 ± 3.6	12.8 ± 3.1	0.704
Creatinine (mg/dL)	0.8 ± 0.2	0.8 ± 0.3	0.283
Uric acid (mg/dL)	5.8 ± 1.4	5.7 ± 1.5	0.582
Na (mEq/L)	141.1 ± 2.2	140.2 ± 2.1	0.447
K (mEq/L)	4.2 ± 0.4	4.2 ± 0.3	0.966
u-Microalbumin (*μ*g/mL)	23.9 ± 43.9	76.1 ± 113.7	0.375
Ferritin (ng/mL)	184.4 ± 167.9	159.9 ± 130.6	0.767
Creatine phosphokinase (IU/L)	108.2 ± 44.1	124.4 ± 80.4	0.899
HOMA-IR	3.55 ± 1.68	3.84 ± 2.30	0.865
HOMA-*β*	98.1 ± 71.2	84.5 ± 53.6	0.865
Complete blood count			
White blood cells (×10^3^/*μ*L)	6.6 ± 1.2	5.4 ± 0.9	0.052
Hemoglobin (g/dL)	14.7 ± 1.4	14.9 ± 1.3	0.865
Platelets (×10^3^/*μ*L)	23.6 ± 5.4	20.1 ± 4.6	0.163
Inflammatory markers			
Hyaluronic acid (ng/mL)	38.3 ± 29.1	57.5 ± 46.2	0.446
7S domain of type 4 collagen (ng/mL)	4.4 ± 0.7	4.4 ± 0.9	0.445
High-sensitivity CRP (mg/dL)	0.131 ± 0.099	0.130 ± 0.122	0.964
Procollagen-3-peptide (U/mL)	0.57 ± 0.09	0.51 ± 0.05	0.175
Zn (*μ*g/dL)	88.8 ± 13.9	88.0 ± 17.1	0.612
Total adiponectin (*μ*g/mL)	7.3 ± 1.5	7.8 ± 1.1	0.400
Interleukin-6 (pg/mL)	3.4 ± 5.5	1.7 ± 1.0	0.309
Bile acids (BA)			
Total BA (*μ*mol/L)	2.51 ± 1.82	5.34 ± 4.87	0.331
Primary BA (*μ*mol/L)	1.32 ± 1.74	2.99 ± 3.16	0.135
Secondary BA (*μ*mol/L)	1.18 ± 0.97	2.36 ± 3.41	0.966
CT scan			
Visceral fat (cm^2^)	188.8 ± 73.7	170.8 ± 51.1	0.673
Subcutaneous fat (cm^2^)	257.1 ± 150.4	252.9 ± 76.1	0.877
V/S ratio	0.92 ± 0.57	0.71 ± 0.26	0.612
CT-L1 (HU)	39.9 ± 10.5	50.2 ± 11.9	0.025
CT-L2 (HU)	40.2 ± 13.8	50.7 ± 11.3	0.063
CT-Spleen (HU)	49.2 ± 2.9	49.9 ± 4.7	0.866
L/S ratio	0.82 ± 0.25	1.01 ± 0.23	0.035
Baseline treatment for hypertension [*n* (%)]			0.973
Naive	5 (41.7)	3 (42.9)	
Other ARB or ACE inhibitor	3 (25.0)	2 (28.6)	
Calcium channel blocker	4 (33.3)	2 (28.6)	
Baseline treatment for diabetes mellitus [*n* (%)]			0.123
Diet only	12 (100.0)	5 (71.4)	
Sulphonylurea+*α*-glucosidase inhibitor	0 (0.0)	2 (28.6)	
Baseline treatment for lipid abnormality [*n* (%)]			0.603
Diet only	10 (83.3)	5 (71.4)	
HMG-CoA reductase inhibitor (statin)	2 (16.7)	2 (28.6)	

Data are mean ± SD. Parameters were compared between groups (telmisartan versus losartan) by Mann-Whitney *U* test or chi-squared test.

**Table 2:** Changes in anthropometric measurements and blood pressure.

Parameters	Group	0 month	12 months	*P*-value	Difference	*P*-value
Body mass index (kg/m^2^)	T	29.2 ± 5.8	29.0 ± 5.9	0.875	−0.2 ± 1.1	
	L	27.8 ± 3.8	28.1 ± 4.2	0.398	0.3 ± 0.8	0.447
Waist circumference (cm)	T	97.0 ± 14.9	98.2 ± 15.5	0.247	1.3 ± 3.4	
	L	94.4 ± 8.1	99.6 ± 8.6	0.091	5.3 ± 7.8	0.310
Hip circumference (cm)	T	111.9 ± 20.9	107.9 ± 15.0	0.500	−2.1 ± 8.3	
	L	99.6 ± 8.7	98.3 ± 9.1	0.655	−0.1 ± 0.4	0.754
Systolic blood pressure (mmHg)	T	139.4 ± 11.1	130.8 ± 15.0	0.045	−8.6 ± 15.2	
	L	136.4 ± 13.9	127.4 ± 10.6	0.046	−9.0 ± 10.4	0.933
Diastolic blood pressure (mmHg)	T	86.0 ± 8.5	75.4 ± 12.7	0.032	−10.6 ± 13.7	
	L	81.6 ± 13.0	75.3 ± 7.9	0.116	−6.3 ± 9.1	0.446
Pulse (beats/min)	T	74.5 ± 11.8	73.2 ± 9.4	0.397	−1.2 ± 5.8	
	L	85.0 ± 9.5	76.4 ± 11.5	0.273	−5.0 ± 10.2	0.397

Data are presented as mean ± SD. Parameters at 0 and 12 months of treatment were compared by Wilcoxon's matched-pair signed-rank test. Differences are shown as [value at 12 months − value at 0 month]. Differences between groups (telmisartan (T) versus losartan (L)) were compared by Mann-Whitney *U* test.

**Table 3:** Changes in biochemical measurements.

Parameters	Group	0 month	12 months	*P*-value	Difference	*P*-value
Aspartate aminotransferase (IU/L)	T	30.6 ± 13.9	35.3 ± 19.0	0.583	4.8 ± 17.6	
	L	32.0 ± 10.3	32.3 ± 12.3	>0.999	0.3 ± 5.6	0.672
Alanine aminotransferase (IU/L)	T	39.8 ± 26.6	50.3 ± 32.3	0.261	10.5 ± 28.3	
	L	43.7 ± 26.2	46.4 ± 28.7	0.344	2.7 ± 8.6	0.672
*γ* Glutamyl transpeptidase (IU/L)	T	58.9 ± 43.0	69.2 ± 72.7	0.683	10.3 ± 56.8	
	L	60.9 ± 63.8	57.6 ± 57.8	0.463	−3.3 ± 9.2	0.471
Total cholesterol (mg/dL)	T	217.8 ± 42.3	212.4 ± 29.7	0.505	−5.3 ± 19.8	
	L	201.0 ± 38.9	198.9 ± 31.8	0.345	−2.1 ± 23.8	0.525
Triglyceride (mg/dL)	T	122.3 ± 54.3	128.5 ± 55.1	0.433	6.2 ± 54.3	
	L	120.9 ± 45.7	122.0 ± 50.9	0.866	1.1 ± 31.8	0.899
HDL cholesterol (mg/dL)	T	52.8 ± 13.1	51.1 ± 12.9	0.283	−1.8 ± 4.8	
	L	46.7 ± 9.2	45.7 ± 11.0	0.343	−1.0 ± 3.1	>0.999
Free fatty acids (mEq/L)	T	0.87 ± 0.26	0.59 ± 0.22	0.005	−0.28 ± 0.27	
	L	0.50 ± 0.26	0.66 ± 0.22	0.237	0.16 ± 0.29	0.007
Fasting plasma glucose (mg/dL)	T	116.5 ± 20.3	112.3 ± 11.1	0.247	−4.2 ± 15.5	
	L	122.8 ± 20.2	137.0 ± 28.6	0.078	14.2 ± 12.9	0.031
IRI (*μ*U/mL)	T	12.5 ± 6.1	14.5 ± 11.1	0.720	2.1 ± 7.5	
	L	12.6 ± 6.4	10.2 ± 3.8	0.498	−2.4 ± 4.8	0.396
Hemoglobin A1c (%)	T	6.4 ± 0.6	6.4 ± 0.4	0.552	0.0 ± 0.4	
	L	6.7 ± 1.0	7.2 ± 1.2	0.017	0.5 ± 0.2	0.001
HOMA-IR	T	3.55 ± 1.68	3.98 ± 2.92	>0.999	0.43 ± 2.45	
	L	3.84 ± 2.30	3.43 ± 1.46	0.686	−0.41 ± 1.63	0.770
HOMA-*β*	T	98.1 ± 71.2	115.5 ± 103.6	0.374	17.4 ± 42.8	
	L	84.5 ± 53.6	58.5 ± 33.6	0.043	−26.0 ± 29.6	0.062
Creatinine (mg/dL)	T	0.8 ± 0.2	0.9 ± 0.2	0.149	0.03 ± 0.06	
	L	0.8 ± 0.3	0.8 ± 0.3	0.655	0.00 ± 0.06	0.274
Uric acid (mg/dL)	T	5.8 ± 1.4	6.3 ± 1.2	0.016	0.4 ± 0.5	
	L	5.7 ± 1.5	5.2 ± 1.3	0.046	−0.5 ± 0.5	0.002
Primary bile acids (*μ*mol/L)	T	1.3 ± 1.7	2.0 ± 2.3	0.534	0.7 ± 3.0	
	L	3.0 ± 3.2	2.8 ± 2.6	0.345	−0.2 ± 1.6	0.447
Secondary bile acids (*μ*mol/L)	T	1.2 ± 1.0	1.4 ± 1.8	0.906	0.2 ± 1.3	
	L	2.4 ± 3.4	2.4 ± 2.5	0.735	0.0 ± 2.1	0.554
Microalbumin in urine (*μ*g/mL)	T	23.9 ± 43.9	24.5 ± 46.6	0.173	−0.5 ± 53.0	
	L	76.1 ± 113.7	39.5 ± 46.2	0.173	−36.6 ± 58.5	0.884

Data are presented as mean ± SD. Parameters at 0 and 12 months of treatment were compared by Wilcoxon's matched-pair signed-rank test. Differences are shown as [value at 12 months − value at 0 month]. Differences between groups (telmisartan (T) versus losartan (L)) were compared by Mann-Whitney *U* test.

HDL: high-density lipoprotein, IRI: immunoreactive insulin, and HOMA-IR: homeostasis model assessment-insulin resistance.

**Table 4:** Changes in inflammatory markers.

Parameters	Group	0 month	12 months	*P*-value	Difference	*P*-value
Hyaluronic acid (ng/mL)	T	38.3 ± 29.1	51.9 ± 41.3	0.091	13.6 ± 23.9	
	L	57.5 ± 46.2	60.1 ± 44.5	0.345	2.6 ± 5.8	0.310
7S domain of type 4 collagen (ng/mL)	T	4.4 ± 0.7	4.5 ± 1.7	0.723	0.07 ± 1.46	
	L	4.4 ± 0.9	4.4 ± 0.9	0.834	0.03 ± 0.44	0.419
High-sensitivity C-reactive protein (mg/dL)	T	0.13 ± 0.10	0.20 ± 0.14	0.155	0.09 ± 0.15	
	L	0.13 ± 0.12	0.11 ± 0.09	0.176	−0.02 ± 0.04	0.077
Procollagen-3-peptide (U/mL)	T	0.57 ± 0.09	0.57 ± 0.13	0.656	−0.003 ± 0.134	
	L	0.51 ± 0.05	0.49 ± 0.11	0.351	−0.023 ± 0.098	0.766
Zinc (*μ*g/dL)	T	88.8 ± 13.9	85.1 ± 17.7	0.139	−3.8 ± 7.8	
	L	88.0 ± 17.1	89.1 ± 15.6	0.735	1.1 ± 12.3	0.374
Total adiponectin (*μ*g/mL)	T	7.3 ± 1.5	7.1 ± 2.1	0.553	−0.2 ± 0.9	
	L	7.8 ± 1.1	8.3 ± 1.5	0.753	0.5 ± 2.1	0.561
Interleukin-6 (pg/mL)	T	3.4 ± 5.5	2.5 ± 0.8	0.158	−0.9 ± 5.9	
	L	1.7 ± 1.0	2.3 ± 1.8	0.173	0.6 ± 1.0	0.766

Data are presented as mean ± SD. Parameters at 0 and 12 months of treatment were compared by Wilcoxon's matched-pair signed-rank test. Differences are shown as [value at 12 months − value at 0 month]. Differences between groups (telmisartan (T) versus losartan (L)) were compared by Mann-Whitney *U* test.

**Table 5:** Changes in fat distribution on CT scanning.

Parameters	Group	0 month	12 months	*P*-value	Difference	*P*-value
Visceral fat area (cm^2^)	T	188.8 ± 73.7	188.8 ± 92.3	0.695	0.0 ± 47.5	
	L	170.8 ± 51.1	181.5 ± 23.6	0.345	10.7 ± 35.5	0.353
Subcutaneous fat area (cm^2^)	T	257.1 ± 150.4	252.7 ± 171.5	0.875	−4.4 ± 52.0	
	L	252.9 ± 76.1	253.8 ± 65.5	0.834	0.9 ± 27.2	0.933
Visceral to subcutaneous fat ratio	T	0.92 ± 0.57	1.12 ± 1.02	0.754	0.19 ± 0.74	
	L	0.71 ± 0.26	0.75 ± 0.19	0.176	0.05 ± 0.15	0.398
Liver to spleen ratio	T	0.82 ± 0.25	0.97 ± 0.22	0.049	0.16 ± 0.24	
	L	1.01 ± 0.23	1.01 ± 0.21	>0.999	0.00 ± 0.16	0.272

Data are presented as mean ± SD. Parameters at 0 and 12 months of treatment were compared by Wilcoxon's matched-pair signed-rank test. Differences are shown as [value at 12 months − value at 0 month]. Differences between groups (telmisartan (T) versus losartan (L)) were compared by Mann-Whitney *U* test.

CT: computer tomography.
