# Erratum to “Effect of Telmisartan or Losartan for Treatment of Nonalcoholic Fatty Liver Disease: Fatty Liver Protection Trial by Telmisartan or Losartan Study (FANTASY)”

**DOI:** 10.1155/2014/302602

**Published:** 2014-04-27

**Authors:** Takumi Hirata, Kengo Tomita, Toshihide Kawai, Hirokazu Yokoyama, Akira Shimada, Masahiro Kikuchi, Hiroshi Hirose, Hirotoshi Ebinuma, Junichiro Irie, Keisuke Ojiro, Yoichi Oikawa, Hidetsugu Saito, Hiroshi Itoh, Toshifumi Hibi

**Affiliations:** ^1^Department of Internal Medicine, School of Medicine, Keio University, 35 Shinanomachi, Shinjuku-ku, Tokyo 160-8582, Japan; ^2^Department of Internal Medicine, National Defense Medical College, 3-2 Namiki, Tokorozawa-Shi, Saitama 359-8513, Japan

In the paper, the dosage of telmisartan is incorrect due to a typographical error. Twelve patients, assigned to telmisartan group, received telmisartan at a dose of 40 mg, not 20 mg, once a day. The correct sentences should appear as given below.

1. “Nineteen hypertensive NAFLD patients with type 2 diabetes were randomly assigned to receive telmisartan at a dose of 40 mg once a day (*n* = 12) or losartan at a dose of 50 mg once a day (*n* = 7) for 12 months” in the “Methods” section of the “Abstract.”
2. “Nineteen hypertensive NAFLD patients with type 2 diabetes were randomly assigned to the telmisartan (T) group (receiving a standard dose of 40 mg once daily, *n* = 12) or the losartan (L) group (receiving a standard dose of 50 mg once daily, *n* = 7)” in Section 2.2.

